# Successful treatment with baloxavir marboxil of a patient with peramivir-resistant influenza A/H3N2 with a dual E119D/R292K substitution after allogeneic hematopoietic cell transplantation: a case report

**DOI:** 10.1186/s12879-020-05205-1

**Published:** 2020-07-06

**Authors:** Naonori Harada, Wataru Shibata, Hideo Koh, Emi Takashita, Seiichiro Fujisaki, Hiroshi Okamura, Satoru Nanno, Koichi Yamada, Hirohisa Nakamae, Masayuki Hino, Hiroshi Kakeya

**Affiliations:** 1 Hematology, Graduate School of Medicine, Osaka City University, 1-4-3 Asahi-machi, Abeno-ku, Osaka, 545-8585 Japan; 2 Department of Infection Control Science, Graduate School of Medicine, Osaka City University, Osaka, Japan; 3 Research Center for Infectious Disease Sciences (RCIDS), Graduate School of Medicine, Osaka City University, Osaka, Japan; 4 Influenza Virus Research Center, National Institute of Infectious Diseases, Tokyo, Japan

**Keywords:** Allogeneic hematopoietic cell transplantation, Baloxavir marboxil, Neuraminidase mutation, Dual E119D/R292K substitution, Peramivir resistance, Immunocompromised host, Influenza A/H3N2

## Abstract

**Background:**

Extended use of oseltamivir in an immunocompromised host could reportedly induce neuraminidase gene mutation possibly leading to oseltamivir-resistant influenza A/H3N2 virus. To our knowledge, no report is available on the clinical course of a severely immunocompromised patient with a dual E119D/R292K neuraminidase mutated-influenza A/H3N2 during the administration of peramivir.

**Case presentation:**

A 49-year-old male patient was admitted for second allogeneic hematopoietic cell transplantation for active acute leukemia. The patient received 5 mg prednisolone and 75 mg cyclosporine and had severe lymphopenia (70/μL). At the time of hospitalization, the patient was diagnosed with upper tract influenza A virus infection, and oseltamivir treatment was initiated immediately. However, the patient was intolerant to oseltamivir. The following day, treatment was changed to peramivir. Despite a total period of neuraminidase-inhibitor administration of 16 days, the symptoms and viral shedding continued. Changing to baloxavir marboxil resolved the symptoms, and the influenza diagnostic test became negative. Subsequently, sequence analysis of the nasopharyngeal specimen revealed the dual E119D/R292K neuraminidase mutant influenza A/H3N2.

**Conclusions:**

In a highly immunocompromised host, clinicians should take care when peramivir is used for extended periods to treat influenza virus A/H3N2 infection as this could potentially leading to a dual E119D/R292K substitution in neuraminidase protein. Baloxavir marboxil may be one of the agents that can be used to treat this type of mutated influenza virus infection.

## Background

In patients undergoing allogeneic hematopoietic cell transplantation (allo-HCT), influenza virus infection carries a potentially fatal risk; in cases of progression from the upper to the lower respiratory tract, the mortality rate of influenza virus infection has been reported to be up to approximately 25% [1]. Risk factors for fatal outcome in leukemia and allo-HCT patients when developing seasonal influenza include influenza soon after chemotherapy, early infection after allo-HCT, lymphopenia, and the lack of early antiviral therapy [2]. In addition, when patients undergoing allo-HCT developed 2009 influenza A/H1N1, neutropenia, older age, long-term steroid use (≥ 20 mg/day of prednisone equivalent), and isolation of an oseltamivir-resistant strain of the 2009 influenza A/H1N1 were reported as risk factors for fatal outcome [2]. European guidelines for influenza in HCT and leukemia patients recommend oseltamivir as the first-line treatment; the alternative treatment is inhaled zanamivir [2]. When the above agents are unavailable, intravenous peramivir is an alternative option in severe influenza [2]. Furthermore, intravenous zanamivir is also an experimental option although it has not yet updated in treatment guidelines. However, in patients undergoing allo-HCT, under treatment with oseltamivir, influenza viral shedding reportedly lasts for a median of 12 days [3]; for up to  48 days [4]. This excretion duration could easily be extended according to the host’s immune status, including severe lymphopenia and higher doses of steroids [3]. In addition, in compromised hosts, long-term use of oseltamivir could result in oseltamivir resistance including via neuraminidase (NA) gene mutations [5]. Hence, clinicians, especially transplant doctors, need various agents other than NA inhibitors for prolonged influenza viral shedding in patients under severe immunosuppression, such as those receiving transplant and those using high amounts of or multiple immunosuppressants.

Here, we demonstrate that baloxavir marboxil (hereafter referred to as baloxavir), a new selective inhibitor of influenza cap-dependent endonuclease [6], was effective in a highly immunocompromised patient with peramivir-resistant dual E119D/R292K NA mutated-influenza A/H3N2 after allo-HCT.

## Case presentation

A 49-year-old male patient was admitted in 2018 for a second allo-HCT with human leukocyte antigen (HLA)-haploidentical related donor peripheral blood for active acute myeloid leukemia with myelodysplasia-related changes. Before first HCT, he had been administered 1 course of azacytidine, but the response had been inefficient. Next, he had received idarubicin and low-dose cytarabine. In the first HCT, the patient had received HLA-matched related donor peripheral blood. The patient had achieved neutrophil engraftment at day 12 after the first HCT. On day 90, he had been diagnosed with relapse of primary disease. He had developed neither acute nor chronic graft-versus-host disease. On hospital day 1 (HD1) on March in 2018, 187 days had elapsed after the first allo-HCT. The respiratory rate was 15 times per a minute and the saturation of percutaneous oxygen was 96%. At the time of hospital admission, the patient had low-grade fever, a sore throat, and a cough. No other symptoms were observed. A rapid diagnostic test for influenza virus antigen using a nasopharyngeal swab specimen was positive for influenza A. No household with influenza-symptoms contacted the patient before the onset. However, a few days before admission to our hospital, the patient stayed home temporarily and sometimes went outdoors. Neither the patient nor household received influenza vaccine for the season. Since we did not find suspected influenza pneumonia on chest computed tomography, the patient was diagnosed with influenza A-upper respiratory tract infection.

**Fig. 1:**
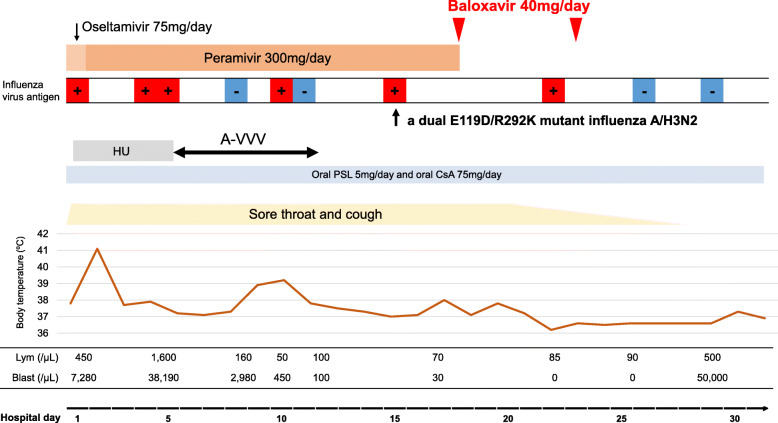
Clinical course in a severely immunosuppressed patient with peramivir-resistant dual E119D/R292K neuraminidase mutated-influenza A/H3N2 after allo-HCT allo-HCT, allogeneic hematopoietic cell transplantation; A-VVV, cytosine arabinoside, etoposide, vincristine, and vinblastine; CsA, cyclosporine A; HU, hydroxyurea; Lym, lymphocyte; PSL, prednisolone

From HD1, treatment with 75-mg oseltamivir twice daily was initiated. However, the patient developed transient hypotension after taking oseltamivir. We were unable to rule out the possibility of the side effect of oseltamivir by clinical judgement. Therefore, the following day, considering the possibility of the intolerance, treatment was changed to intravenous 300 mg-peramivir once daily, since oseltamivir and peramivir, but not inhalant drugs, are recommended for critically ill patients by the Japanese association for infectious diseases [7]. Since the primary disease was highly active with marrow containing mainly blast cells, the patient was treated with oral hydroxyurea on HD1 and a 6-day A-VVV intravenous regimen (cytosine arabinoside, etoposide, vincristine, and vinblastine) on HD6 to reduce the leukemia cells. Despite the continuous anti-influenza combination therapy, the sore throat and cough persisted and low-grade fever did not improve. Moreover, the repeated diagnostic influenza tests were positive (Fig. 1). Considering possible peramivir resistance in combination with the highly immunocompromised host with severe lymphopenia (70/μL) and hypogammaglobulinemia (IgG 765 mg/dL, IgA 61 mg/dL, and IgM 19 mg/dL [normal range; 870–1700 mg/dL, 93–393 mg/dL, and 33–183 mg/dL, respectively]) under treatment with oral 5-mg/day prednisolone and oral 75-mg/day cyclosporine, 40-mg oral baloxavir once daily was administered on HD18 in place of peramivir. Thereafter, the symptoms started improving; however, the diagnostic influenza test remained positive (Fig. 1). After baloxavir was re-administered on HD23, the test turned negative on HD 26, and we confirmed a negative result on HD29. The symptoms suggestive of influenza infection resolved completely; however, the patient died as a result of primary disease progression on HD62 without receiving second HCT.

We examined the HD15-influenza-positive sample by polymerase chain reaction and sequence analysis, which detected a dual E119D/R292K mutant influenza A/H3N2.

## Discussion and conclusions

The patient developed seasonal influenza A/H3N2 infection while in a highly immunocompromised state post-transplant-relapsed leukemia; treatment with 1-day oseltamivir and switched to 15-day peramivir were unable to improve the respiratory symptoms and influenza viral shedding possibly due to peramivir resistance caused by the dual E119D/R292K NA mutation. However, after cessation of peramivir, single-dose baloxavir clearly improved the symptoms, and a second dose of baloxavir 5 days later completely resolved both the symptoms and the viral shedding. Baloxavir could be a potent agent for NA inhibitor-resistant influenza infection in immunocompromised patients.

Experimental data suggest that the emergence of NA-inhibitor resistance in influenza A virus may be explained by two mechanisms: NA-independent and NA-dependent mechanisms [8]. Regarding the former mechanism, mutations in or close to the hemagglutinin receptor-binding site reduce the efficiency of viral binding, leading to diminished dependence on NA function [8]. As for the latter mechanisms, amino acid substitutions at the conserved residues in the NA enzyme active site result in NA resistance [8, 9]. The World Health Organization (WHO) Global Influenza Surveillance and Response System (GISRS) Expert Working Group for Surveillance of Antiviral Susceptibility (WHO-AVWG) has conducted global surveillance to determine the NA inhibitor susceptibility of influenza viruses since the 2012–13 period [10]. In the global surveillance, the frequency of viruses with reduced susceptibility to one or more NA inhibitors has remained low (2012–13: 0.6%; 2013–14: 1.9%; 2014–15: 0.5%; 2015–16: 0.8%; 2016–17: 0.2%), while oseltamivir and peramivir cross-resistant A/H1N1pdm09 viruses with an NA H275Y substitution were the most frequently observed [10–14]. The national antiviral resistance surveillance in Japan [15] found that the frequency of oseltamivir and peramivir cross-resistant A/H1N1pdm09 viruses has also remained low in Japan (2010–11: 2.0%; 2011–12: 0%; 2012–13: 1.8%; 2013–14: 4.1%; 2014–15: 0%; 2015–16: 1.9%; 2016–17: 1.1%; 2017–2018: 1.7%; 2018–2019: 0.9%). Each one oseltamivir and peramivir cross-resistant A/H3N2 virus with the R292K mutation was detected in the 2010–11, 2011–12, and 2014–2015 seasons, respectively [12, 13].

In the present case, a dual E119D/R292K mutant influenza A/H3N2 was found. Zurcher, T et al. reported that the E119D mutation in A/H3N2 showed reduced susceptibility to zanamivir [16], although it has not been detected after zanamivir treatment in vivo. The R292K mutation in A/H3N2 was detected in immunocompromised patients after oseltamivir and/or peramivir treatment. These R292K mutant viruses showed highly reduced inhibition by oseltamivir and peramivir and reduced inhibition by zanamivir, but susceptible to laninamivir [12, 13, 17]. Furthermore, the dual NA inhibitor-resistant mutation showed the synergistic effect on the NA inhibitor susceptibility, resulting in enhanced resistance [18]. On the basis of these observations, the dual E119D/R292K mutation in A/H3N2 could have contributed to reduced susceptibility to oseltamivir, peramivir, and zanamivir. To our knowledge, there is no report on this dual mutation in the NA in H3N2.

Evidence suggests that the emergence of NA-inhibitor resistance could be associated with prolonged use of NA inhibitors in compromised hosts [5]. In the present case, we administered peramivir for 15 days, possibly leading to peramivir resistance.

Baloxavir is a new antiviral drug targeting the polymerase acidic protein, one of the protein subunits of the polymerase heterotrimer of the influenza virus, that has a mechanism of action quite different from that of conventional anti-influenza drugs including NA inhibitors [6]. Randomized, controlled trial data in influenza A- and/or B-infected patients not requiring inpatient treatment showed that although the time to the improvement of symptoms as primary analysis was similar in both the baloxavir and oseltamivir groups, baloxavir was associated with a significantly more rapid decline in viral load than oseltamivir as one of secondary analyses [6]. In addition, a recent Phase 3 Trial of baloxavir reported that baloxavir was superior to oseltamivir in shortening the duration of virus replication in high-risk patients with risk factors such as chronic lung disease and age ≥ 65 years [19]. However, thus far, no study has examined the efficacy of baloxavir for influenza infection in highly immunocompromised patients.

In general, post-allo-HCT patients can develop a range of bacterial, fungal, and/or viral infections due to impaired cellular immunity (e.g. T cells and natural killer cells) and humoral immunity (e.g. B cells and immunoglobulin production), especially within at least 1-year post-allo-HCT [20]. Earlier immune reconstitution is warranted to decrease the risk of infection, since a delayed immune reconstitution could lead to infection-related morbidity and mortality. However, relapsed leukemia may delay the immune recovery, including the recovery of lymphocytes. In addition, steroid and calcineurin inhibitors could suppress the host immune system. In the present case, in addition to these factors, the patient was undergoing chemotherapy during active influenza infection which is a potentially fatal risk factor [2]. Therefore, the present case was considered to be a severely immunocompromised host with a high-risk of mortality. Here, we demonstrated the first case of successful treatment using baloxavir for the dual E119D/R292K NA-mutated peramivir-resistant influenza A/H3N2 in a highly immunocompromised patient.

In conclusion, baloxavir could be an option for peramivir-resistant influenza A/H3N2 infection in immunosuppressed hosts, including allo-HCT recipients.

## Data Availability

Not applicable.

## Abbreviations

allo-HCT: Allogeneic hematopoietic cell transplantation
HD: Hospital day
HLA: Human leukocyte antigen
NA: Neuraminidase
